# 

**Published:** 1854-02

**Authors:** 


☞ The old and enterprising Publishing House of Phillips, Sampson & Co., of Boston, have located a branch in Park Place, New York, conducted by Mr. Derby, who will furnish the publications named on the second page of cover, as also all the other issues of that house.

---

### NOTICES.

Prepayment in all cases is indispensable.

The receipt of the "Journal" *unordered*, will be presumptive evidence that it is sent as a specimen, number, and that it is the special desire of the Editor that the person to whom it is sent should become a subscriber.

The postage of the Journal is only six cents a year, if pre-paid at the Post Office of each subscriber.

☞ All written communications to be addressed to

"DR. W. W. HALL, NEW YORK."

All orders, exchanges, and books for notice or review, must be addressed simply to

"HALL'S JOURNAL OF HEALTH, NEW YORK,"

or, if within the city, to the office of the Editor, 37 Irving Place, New York, near Union Square, until May 1st next, and thereafter, at his private residence, 42 Irving Place.

A single specimen number will be sent by the Editor, post-paid, to his personal friends and former patients in the different states of the Union; and he wishes it to be considered as his particular desire, that such would become subscribers; thus affording him, from time to time, the satisfaction of knowing that they "STILL LIVE," and that his labors are tending to promote the health and consequent happiness of those whom he has once pleasurably known.

One specimen number will be also sent to a number of clergymen, for it originated in a desire to promote their good, and the Editor will endeavor to conduct it in such a manner, that any pains they take to procure subscribers, especially from among the young men and young women of their congregations, will be compensated in the permanent healthfulness of those who are to take their places when their own labor is done, and they have gone up to their reward.

The Journal will be sent also to some of the public men in the country, whose health and length of life are believed to be necessary to the highest interests of the communities in which they reside. The postage on all specimen numbers will be prepaid at the New York Post Office, and it is specially requested, to preserve the Editor from loss, that the specimen number be returned, when not taken, to address of "Hall's Journal of Health, New York."

A PREMIUM of twenty-five dollars will be paid to any subscriber who will write, from his own experience, the best description of How I LOST MY HEALTH, to contain about eight hundred words, on or before the first day of July next. A sealed envelope should accompany the manuscript, containing the full name and address of the author, none of which will be broken, except that of the successful one; these envelopes will be returned by mail, unopened and prepaid, to each one who desires it. The manuscripts will be retained, unless called for.

### CONTENTS OF JANUARY NUMBER.

| | Page | | Page |
|---|---|---|---|
| Editor's Address | 1 | Wearing the Beard | 15 |
| Subjects to be Treated | 5 | Invalids and Exertive Exercise | 16 |
| Eating and Drinking | 6 | Heart Disease | 17 |
| Tea and Coffee | 6 | Consumption and the South | 18 |
| Use of Milk | 7 | Book Notices | 22 |
| Dr. Nott's Munificence | 8 | "The Independent" | 22 |
| The food we Eat | 11 | The Home Journal | 22 |
| How to attain Old Age | 12 | The American Medical Monthly | 23 |
| Too white Flour | 13 | To Correspondents | 23 |
| Traveling for Health | 14 | A Premium | 24 |

### CONTENTS OF FEBRUARY NUMBER.

| | Page | | Page |
|---|---|---|---|
| Invalids and Exertive Exercise | 25 | Horseback Exercise | 40 |
| Common Sense | 27 | Going to the South | 41 |
| Longevity | 30 | Would a glass of Wine hurt me ? | 42 |
| Ventilation | 31 | How to Sleep | 43 |
| Ignorance, fatal effects of | 33 | To Correspondents | 47 |
| Over-working | 33 | A Premium | 48 |
| Air and Exercise | 35 | | |

☞ The "Journal" will be sent one year to any established newspaper or periodical which will notice its appearance, and copy the contents of this month's number—*provided* such publication is sent, with the article scored, to address of

"HALL'S JOURNAL OF HEALTH, NEW YORK."

☞Persons to whom this number is sent, post paid, will please consider it an intimation that the editor desires them to become subscribers.

